# Exocrine-Endocrine Crosstalk: The Influence of Pancreatic Cellular Communications on Organ Growth, Function and Disease

**DOI:** 10.3389/fendo.2022.904004

**Published:** 2022-06-13

**Authors:** Danielle L. Overton, Teresa L. Mastracci

**Affiliations:** ^1^ Department of Biology, Indiana University-Purdue University Indianapolis, Indianapolis, IN, United States; ^2^ Department of Biochemistry and Molecular Biology, Indiana University School of Medicine, Indianapolis, IN, United States; ^3^ Center for Diabetes and Metabolic Diseases, Indiana University School of Medicine, Indianapolis, IN, United States

**Keywords:** beta cell, exocrine pancreas, crosstalk, diabetes, exocrine pancreas dysfunction, pancreas development

## Abstract

Diabetes mellitus, a disease that affects nearly 536.6 million people worldwide, is characterized by the death or dysfunction of insulin-producing beta cells of the pancreas. The beta cells are found within the islets of Langerhans, which are composed of multiple hormone-producing endocrine cells including the alpha (glucagon), delta (somatostatin), PP (pancreatic polypeptide), and epsilon (ghrelin) cells. There is direct evidence that physical and paracrine interactions between the cells in the islet facilitate and support beta cell function. However, communication between endocrine and exocrine cells in the pancreas may also directly impact beta cell growth and function. Herein we review literature that contributes to the view that “crosstalk” between neighboring cells within the pancreas influences beta cell growth and function and the maintenance of beta cell health.

## Introduction

Diabetes mellitus is a chronic condition marked by elevated blood glucose levels, which can result from either the death or dysfunction of the insulin-producing beta cells in the pancreas. As of 2021, the global prevalence of diabetes cases was estimated to be 536.6 million with expectations to reach 783.2 million by 2045 (1). Research investigating how beta cells grow and function in the normal setting has provided a greater understanding of the pathways leading to beta cell dysfunction as well as possible avenues to reverse beta cell-related disease.

The insulin-producing beta cells are found within structures known as the islets of Langerhans, which are composed of multiple endocrine hormone-producing cell types including alpha (glucagon), delta (somatostatin), PP (pancreatic polypeptide), and epsilon (ghrelin) cells. As is made obvious by the complications resulting from diabetes, beta cells perform a function critical for the maintenance of whole-body metabolism. These cells are responsible for the secretion of insulin in response to elevated blood glucose levels, a function necessary to maintain whole-body metabolism. Interestingly, there is significant evidence that physical and paracrine interactions within the pancreas contribute to the maintenance of beta cell function. Moreover, there is mounting evidence that the islets/beta cells are influenced by the surrounding pancreatic exocrine acinar cells [reviewed in (2, 3)], which function to produce and secrete zymogens into the ductal system to facilitate digestion [reviewed in (4)]. Therefore, evaluating how cells within the pancreas communicate and interact can assist our understanding of organ growth, development and function.

The molecular and cellular communications that occur between cells and facilitate biological responses can loosely be categorized as “*crosstalk*”. In the pancreas, the physical proximity of endocrine cells to exocrine cells or to other endocrine cells in the islet, supports the idea that crosstalk may be possible between different pancreatic cell types. Animal models with defects in pancreas development as well as studies of pancreatic pathologies have also assisted our understanding of both pancreatic cell growth, function and dysfunction. Ultimately, investigating the cellular connections within the pancreas in both the healthy and diseased states provides a greater appreciation for the complexity of disease progression and the potential cellular opportunities that could be exploited to slow or reverse dysfunction. In this review, we explore pancreatic cellular crosstalk by examining the developmental cues that can instruct and manipulate certain pancreatic lineage decisions, the role of intra-islet communication and extracellular connections in beta cell function, and how exocrine pancreas function and dysfunction can influence the endocrine pancreas.

## Developmental Signals Dictate Pancreas Form and Function

**Figure 1:**
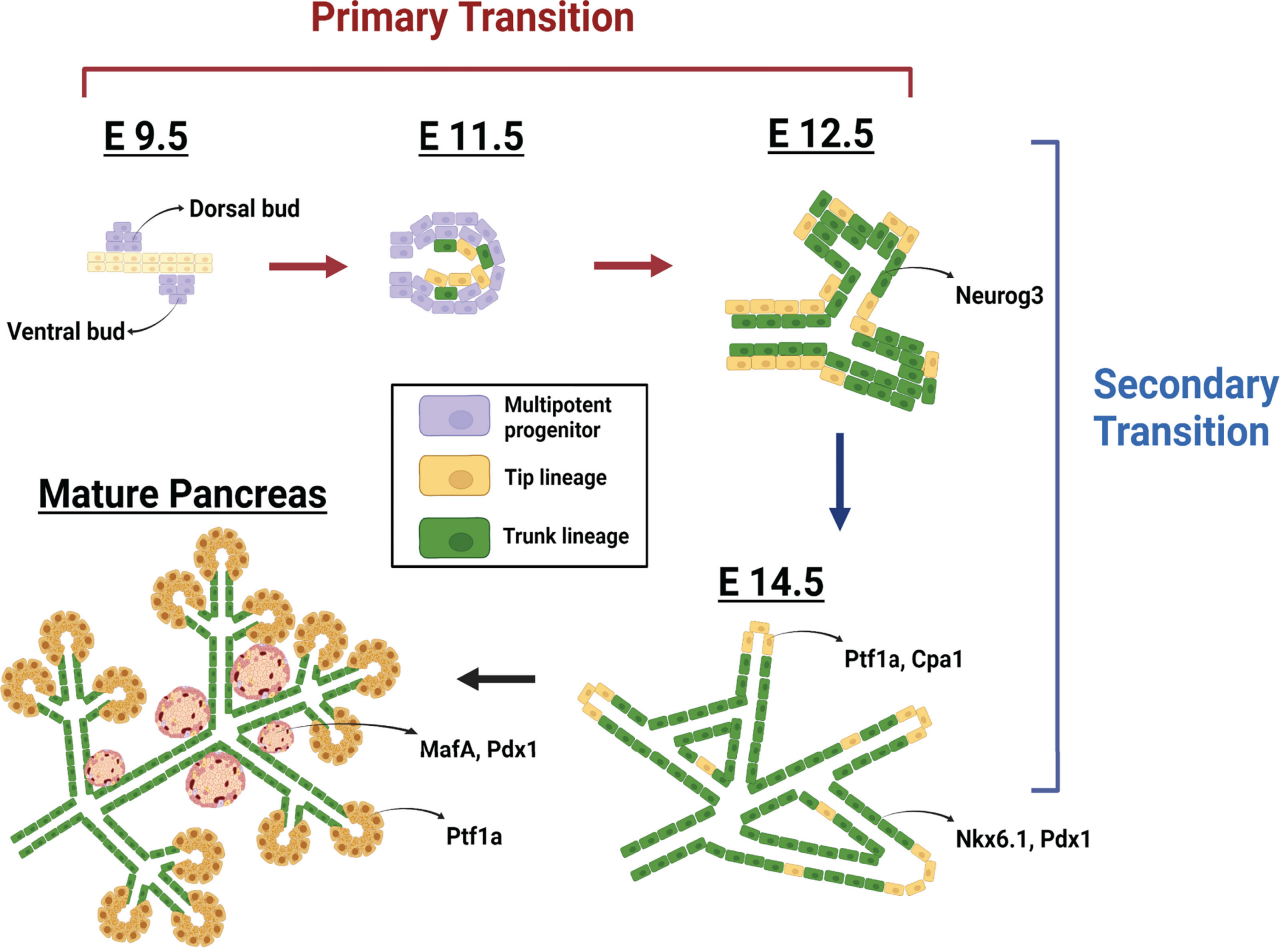
Pancreas development. Embryonic day (E) 9.5 marks the initiation of pancreas development in the mouse and the stage known as the primary transition. This first stage of development is characterized by the emergence of the dorsal and ventral pancreatic buds (purple) from the posterior foregut endoderm. These pancreatic buds are composed of progenitor cells that express the transcription factors Pdx1 and Ptf1a. The subsequent morphogenesis of the pancreatic ducts leads to an organization of the multipotent progenitor cells within the trunk (green) and tip (yellow) domains of the ductal epithelium. During the secondary transition (E12.5 – E15.5), the trunk cells become highly proliferative and push the tip cells to the exterior. Progenitor cells within the trunk express Neurog3 and subsequently differentiate into all islet cell types. Progenitor cells at the ductal tips, expressing Cpa1 and Ptf1a, and differentiate into acinar cells. By birth, the acinar cell mass has expanded and the islet cells begin to coalesce into the structures known as the islets of Langerhans.

Endocrine and exocrine cells develop from a common progenitor cell population (**Figure 1**). In mammals, pancreas development begins when a region of the foregut endoderm receives signals from the aorta, notochord, and surrounding mesenchyme, leading to the emergence of the dorsal pancreatic progenitor cell domain (the “dorsal bud”) (5). Almost simultaneously, another portion of the endoderm receives inductive signals from the cardiac mesoderm, lateral plate mesoderm, and septum transversum mesenchyme to stimulate development of the ventral pancreatic progenitor cell domain (the “ventral bud”) (5). Once the pancreatic domains are specified in the embryo, development proceeds in two distinct stages: the primary transition and the secondary transition (5).

The primary transition begins around embryonic day (E) 9.5 and concludes around E12.5. During this time, the progenitor cells within the dorsal and ventral buds expand by proliferation and organize to form the pancreatic epithelium (5) and the two distinct pancreatic domains fuse to become one organ (5). During the secondary transition, which spans E12.5 to E15.5, progenitor cells within the pancreatic epithelium differentiate, with the exocrine lineage deriving from progenitor cells in the “tip” domain of the epithelium and the endocrine lineage deriving from progenitor cells within the “trunk” domain (5). Changes in the expression of transcription factor networks regulate this pancreatic cellular differentiation, driving the formation of the distinct endocrine and exocrine cell lineages. To that end, transcription factor mutant mouse models have identified many genes responsible for these cell fate decisions. By resolving the factors or mechanisms necessary to instruct cell fate, we have gained significant insight into the cellular interactions that contribute to pancreas and beta cell development and function.

The transcription factors pancreas transcription factor 1 alpha (Ptf1a), pancreatic duodenal homeobox 1 (Pdx1), and NK6 homeobox 1 (Nkx6.1) are critical for the exocrine versus endocrine cell fate decision. Ptf1a is required for the specification and expansion of the pancreatic progenitor cells, as well as their differentiation into acinar cells (6–9). Pdx1 is also necessary for pancreatic organogenesis and then subsequently for proper beta cell development and function. In fact, Pdx1 is considered a master transcriptional regulator in the pancreas due to its roles not only in development but also insulin secretion, cell proliferation, and mitochondrial metabolism (10–14). The co-expression of Pdx1 and Ptf1a specifies the pancreatic progenitor cell pool and defines the initial cellular connection between the endocrine and exocrine pancreas (9). Similar to Pdx1, Nkx6.1 is required for proper development and function of the beta cells and is involved in the transcription factor network that instructs the endocrine versus exocrine fate decision (15–19). Moreover, a relationship between Nkx6 and Ptf1a was discovered wherein these transcription factors function to repress the alternative lineage program, driving the progenitors toward an endocrine or exocrine cell fate, respectively. A study by Schaffer *et al.* (20) demonstrated that changing the timing and localization of expression of Ptf1a and Nkx6.1 altered the differentiation of exocrine and endocrine cells. This study emphasizes the complex gene expression patterns necessary for proper pancreas development. Furthermore, it suggests that transcription factor expression could be exploited in certain circumstances to tip the scale toward increased endocrine cell production as a means to regenerate cells lost as a result of disease.

The creation of mutant animal models has also demonstrated how the exocrine pancreas can be altered. One example of this was shown in the study by Bonal *et al.* (21), which investigated the role of the basic helix-loop-helix protein c-Myc during pancreas development. C-Myc is known to direct progenitor cell expansion and differentiation, and loss of pancreatic c-Myc lead to decreased embryonic pancreas mass and postnatal transdifferentiation of acinar cell into adipocytes (21). The interruption of proper exocrine pancreas development was also observed with genetic deletion of the enzyme deoxyhypusine synthase (*DHPS*) in the embryonic pancreas (22). Loss of pancreatic *Dhps* resulted in a significant reduction in acinar cell mass due to an alteration in the translation of genes critical for exocrine pancreas development and function. The structural changes observed in these two studies alone demonstrate the importance of correct gene expression and signaling pathway integrity for the maintenance of proper pancreatic form and function.

Defining the timing and factors that direct critical lineages decisions in the pancreas can present theoretical opportunities for therapeutic manipulation in the setting of disease. To that end, inducing the expression of certain transcription factors can drive the trans-differentiation of exocrine (acinar) cells or other endocrine cell types into insulin-expressing beta-like cells. Specifically, overexpression of *MafA, Pdx1*, and *Neurog3* redirected acinar cells toward a beta cell-like fate (23). Furthermore, loss of the gene *Arx* in alpha cells was shown to convert these glucagon-expressing cells into functional beta cells (24, 25). Similarly, pancreatic delta cells were manipulated to show the beta cell characteristic of insulin secretion, and thus aid in diabetes recovery, due to the action of FOXO1 (26). Whereas the goal to target a specific endogenous cell population to direct therapeutic trans-differentiation in the setting of diabetes has not yet been achieved, these studies have greatly assisted our understanding of the genes that regulate pancreatic cell growth. Further refining the cellular interactions that contribute to pancreas and beta cell development and function can also come from studying the islets themselves. By examining intra-islet cell communication and the external influences on the beta cells we obtain a greater understanding of the functional and dysfunctional beta cell.

## Direct and Indirect Cellular Communication Contribute to Pancreas Function

Functionally, the endocrine and the exocrine compartments of the pancreas work in concert to achieve metabolic homeostasis. Succinctly, the endocrine cells, housed in the islets of Langerhans, function to secrete hormones that regulate glucose homeostasis and the exocrine cells function to secrete zymogens that become the digestive enzymes in the intestine required for digestion (27). As mentioned above, the physical location of the islets within the exocrine pancreas facilitates possible crosstalk between these cell types. Similarly, the organization of the islets of Langerhans, which bring all of the hormone-producing endocrine cells (alpha, beta, delta, PP and epsilon cells) into contact with each other, facilitates communication and thus there exists the possibility for positive and negative impact on cellular function. In particular, understanding intra-islet paracrine signaling in the developing and postnatal pancreas as well as the interactions of islets with the surrounding extracellular matrix (ECM) provides an opportunity to determine how these signals impact pancreatic beta cell function and/or contribute to beta cell dysfunction.

### Alpha-Beta Cell Communication

**Figure 2:**
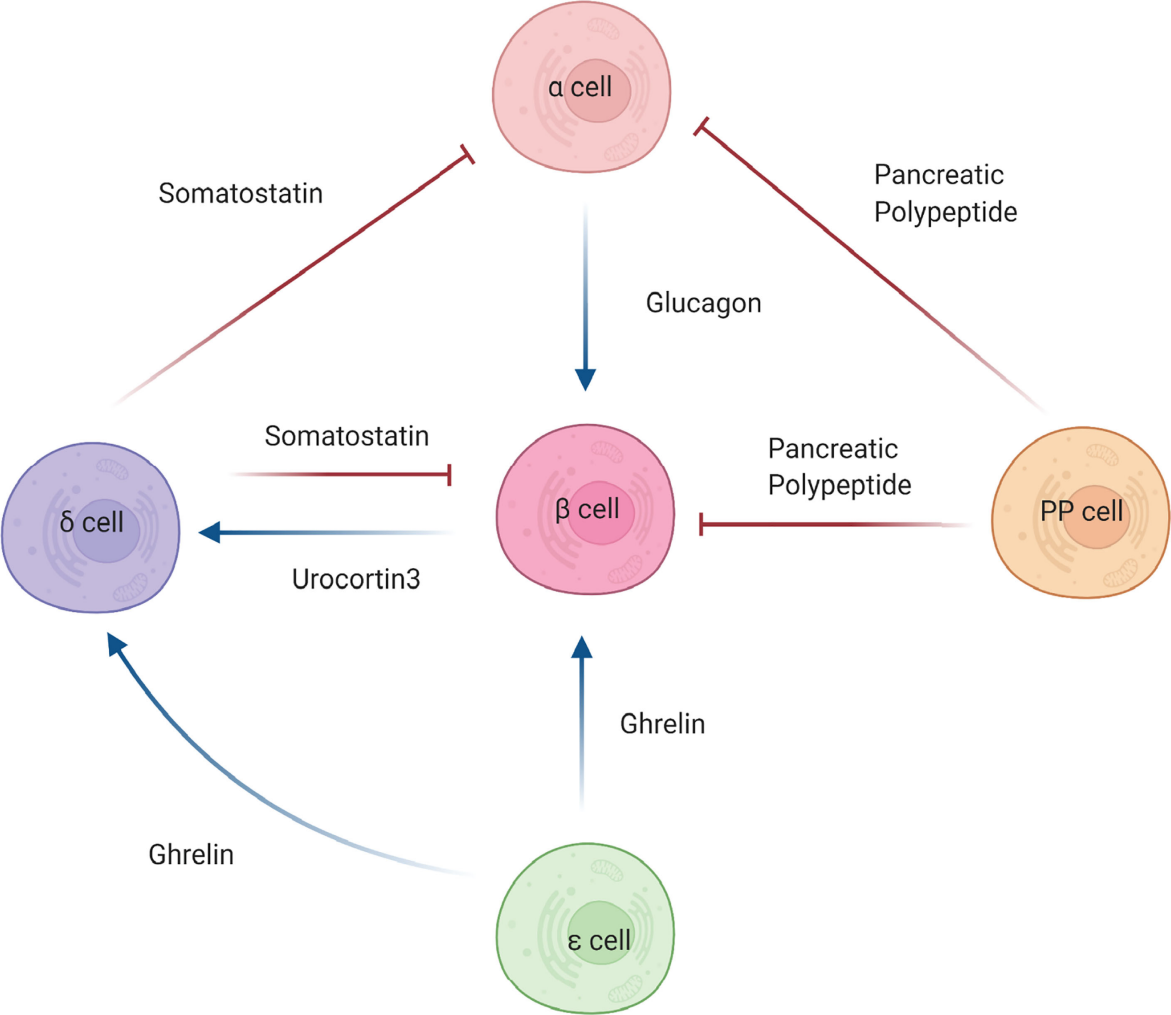
Intra-islet cell communications. Beta (β) cell function is influenced by the actions of other pancreatic islet cells. Alpha cells (α) secrete glucagon, which antagonistically effects insulin secretion from the beta cells. The action of glucagon can can be blunted by somatostatin from delta cells (δ) or pancreatic polypeptide by PP cells, leading to increased insulin secretion. Somatostatin and pancreatic polypeptide can also directly affect beta cells by negatively regulating insulin secretion. Ghrelin, secreted by epsilon cells (ε), positively regulates somatostatin release, which in turn negatively regulates insulin secretion. Furthermore, ghrelin can directly bind beta cells and, in certain forms, promote secretion of insulin. Urocortin3, secreted from the beta cells can mediates insulin secretion via somatostain. Blue arrows denote stimulatory hormonal effects. Red blunted arrows denote inhibitory hormonal effects.

The communication that exists between the alpha and beta cells is commonly described as both counterregulatory and antagonistic, establishing a relationship wherein alpha cells are essential to beta cell function (**Figure 2**). This stems from the fact that beta cells are active during times of hyperglycemia whereas alpha cells are active during times of hypoglycemia [reviewed in (28)]. In essence, the hormones secreted from the alpha and beta cells oppose one another to maintain glucose homeostasis. However, this view of alpha and beta cell communication does not encompass the entirety of their relationship. Beyond their counterregulatory association, alpha and beta cells communicate with one another *via* paracrine signaling in order to maintain functionality. Svendsen *et al.* how insulin secretion is dependent upon intra-islet glucagon signaling. Specifically, beta cells that lack both the glucagon and GLP-1 receptors secrete significantly reduced levels of insulin, suggesting that paracrine signaling from alpha cells is necessary for proper beta cell function (29). Conversely, Kawamori *et al.* showed that deletion of the insulin receptor on alpha cells resulted in glucose intolerance, hyperglycemia, and hyperglucagonemia in male mice during the fed state, suggesting that paracrine signaling from beta cells is necessary for alpha cell function (30). Despite these studies, the hormonal interconnection between glucagon (alpha cells) and insulin (beta cells) remains an open question. Understanding the intricacies for how other secreted factors from these cells affect islet cell function may provide an additional endogenous avenue to positively impact beta cell functional integrity.

### Delta-Beta Cell Communication

Alpha and beta cells also interact closely with another endocrine cell type: delta cells. Delta cells primarily secrete somatostatin, which has been identified as a very strong inhibitor of both alpha cell-mediated glucagon release and beta cell-mediated insulin release (31) (**Figure 2**). Impaired secretion of somatostatin by delta cells has been shown to produce an exaggerated insulin release suggesting that beta cells work concurrently with delta cells *via* paracrine crosstalk to achieve glycemic control (32). The loss of the somatostatin receptor 2 (SSTR2) has been demonstrated to cause significantly higher levels of glucagon secretion, which further supports the necessity for delta cells in the regulation of alpha and beta cell function (33). Interestingly, delta cells were also discovered to express the ghrelin-receptor, which permits communication with ghrelin-expressing epsilon cells thus promoting an inhibitory feedback signal on insulin release (34, 35). Delta cells directly communicate with alpha, beta, and PP cells to maintain glucose homeostasis, showcasing their underrecognized importance and contribution to islet and beta cell function (36). Further investigation of the effects of somatostatin and other delta cell-derived factors is needed to define more completely the intra-islet signaling components emanating from the delta cells that influence beta cell function.

### PP-Beta Cell Communication

An even more underappreciated cell type in the islet is the pancreatic polypeptide-producing PP cells. Pancreatic polypeptide acts primarily as a digestive enzyme, inhibiting acid secretion and generally regulating digestive pancreatic secretions (37). However, the additional role PP cells may play in glucose metabolism is now becoming recognized (**Figure 2**). PP cells have been shown to be not as distantly related to the rest of the islet cell types as once thought (38). Rather, these cells have the potential to secrete hormones other than pancreatic polypeptide including insulin, glucagon and somatostatin, and exhibit the same identity markers as other islet cell types such as beta cells, alpha cells, and delta cells (38). Most excitingly, these cells display the ability to engage in insulin secretion in instances where the beta cells of the islet were damaged, despite originally lacking beta cell identity (38). This discovery connects back to a topic discussed above – the specific circumstances necessary to induce trans-differentiation and whether this can be harnessed to reverse the cellular dysfunction that results from disease. A potential caveat, however, lies in PP cell abundance, as they account for less than 1% of the total islet mass in the pancreatic tail but compose 70% of the total islet mass in the uncinate region and pancreatic head (39). As observed by Furuyama *et al.* (25), directing PP cells to function as insulin-secreting beta cells would also provide a potential therapeutic option in the setting of diabetes.

### Epsilon-Beta Cell Communication

Epsilon cells are the fifth islet cell type found within the islets of Langerhans, although these ghrelin-expressing endocrine cells are found almost exclusively in the embryonic pancreas (40). Previous to their discovery in the pancreas, ghrelin-expressing cells were only identified in the stomach, where they function to secrete ghrelin, “the hunger hormone”, to increase appetite (41). Whereas there are limited studies of the pancreas, ghrelin has been shown to increase blood glucose levels while simultaneously decreasing plasma insulin levels (42) (**Figure 2**). Furthermore, it has been found that the loss of the ghrelin gene (*Ghrl*) in pancreatic epsilon cells has resulted in a significant increase in glucose-stimulated insulin secretion (43). Whereas ghrelin negatively impacts beta cell insulin secretion, in a context dependent manner, it has also been shown to positively impact islet cell mass by promoting cellular proliferation and growth (44). Granata *et al.* (45) demonstrated that in streptozotocin-treated rats, ghrelin gene products increased beta cell survival and prevented diabetes onset. Beta cells and other islet cells possess a ghrelin receptor known as GSH-R1a, through which ghrelin exerts its effects (46). Commonly denoted as the active form, acylated ghrelin specifically utilizes this binding site to activate the GTP-binding protein Gai2, which inhibits cAMP signaling and calcium influx. The result is less insulin release and an increase in blood glucose levels (42, 47). Its counterpart, un-acylated ghrelin, has not been shown to impact insulin sensitivity and is incapable of utilizing the GSH-R1a receptor, therefore demonstrating that it is not active in glucose metabolism (48). However, when acylated ghrelin and un-acylated ghrelin are combined, the result is increased insulin sensitivity, bringing into question the mechanisms by which this occurs (49). Obestatin, the third ghrelin gene product, is the most structurally distinct but arguably the most relevant to beta cell function. It has been shown to inhibit the apoptosis of beta cells, promote insulin release, and aid in sustaining beta cell longevity (50). Most notably, obestatin may serve as a regenerative stimulus for insulin-secreting cells derived from mesenchymal stem cells (51, 52). These studies showcase a positive effect of ghrelin and its derivatives on beta cell function and mass and support the idea that intra-islet derived factors exist that provide a direct benefit to beta cell health.

Although ghrelin alone is able to exert direct effects on beta cells, it should be noted that delta cells are also important in mediating the effect of ghrelin on insulin secretion. Transcriptomic profiling of pancreatic islet cells revealed that the ghrelin receptor gene was highly expressed and enriched in delta cells (35). Furthermore, activation of the ghrelin receptor on delta cells resulted in increased somatostatin secretion concomitant with decreased insulin secretion (35). This study confirms that there exists a complex endocrine regulatory network that is essential for proper insulin secretion and whole-body metabolism.

### Extracellular Influences

In addition to intra-islet communications, islet cells are physically connected with and therefore influenced by their surrounding environment. In particular, the extracellular connections that directly impact islet cell function include (but are not limited to) cell adhesion molecules (CAMs), calcium-dependent adhesion molecules (cadherins), integrins, and connexins (53). Whereas CAMs and cadherins act to ensure cell adhesion, connexins function to allow the transport of cytosolic molecules between connected cells [reviewed in (54)]. Furthermore, integrins act as a scaffold that connects beta cells, and other islet cells, to the extracellular basal lamina in order to receive internal and external signals. Specifically in beta cells, integrins play a direct role in glucose-stimulated insulin secretion by facilitating sensitivity to the presence of glucose (55), and the presence of integrins along with the ECM has been correlated with an increased rate of insulin secretion (56–58). Beta cells also exhibit direct cell-to-cell communication with their fellow islet cells *via* CAMs, which permit molecular communication between adjacent cells. For the mouse islet, cadherins, a subtype of CAMs mediated by calcium signaling, serve to arrange the cells in a distinct layout, with beta cells in the core and other islet cell types composing the mantle (59). In humans, cadherins play a similar structural role while also serving to protect beta cells from apoptosis by maintaining viability (60). It Is also known that beta cells interact with alpha cells by way of E-cadherin, a protein known to be integral to alpha and beta cell communication. To that end, in a beta cell-specific E-cadherin (*CDH1*) mutant animal model, the beta cells were unable to properly cluster and therefore did not receive regulatory signaling from alpha cells leading to disrupted glucose homeostasis (61). In general, this collective group of extracellular connections, which facilitate islet cell communication, may represent a possible avenue to manipulate hormone (e.g. insulin) production and possibly resolve beta cell dysfunction.

In general, the influence on the beta cell of other islet cell types or extracellular connections can be directly linked to physical proximity. With that in mind, the organization of the pancreas as a whole is such that the islets are embedded within the exocrine. Therefore, does form beget function? Can these two functionally distinct compartments directly influence their respective growth and function?

## Exocrine-Endocrine Crosstalk

**Figure 3:**
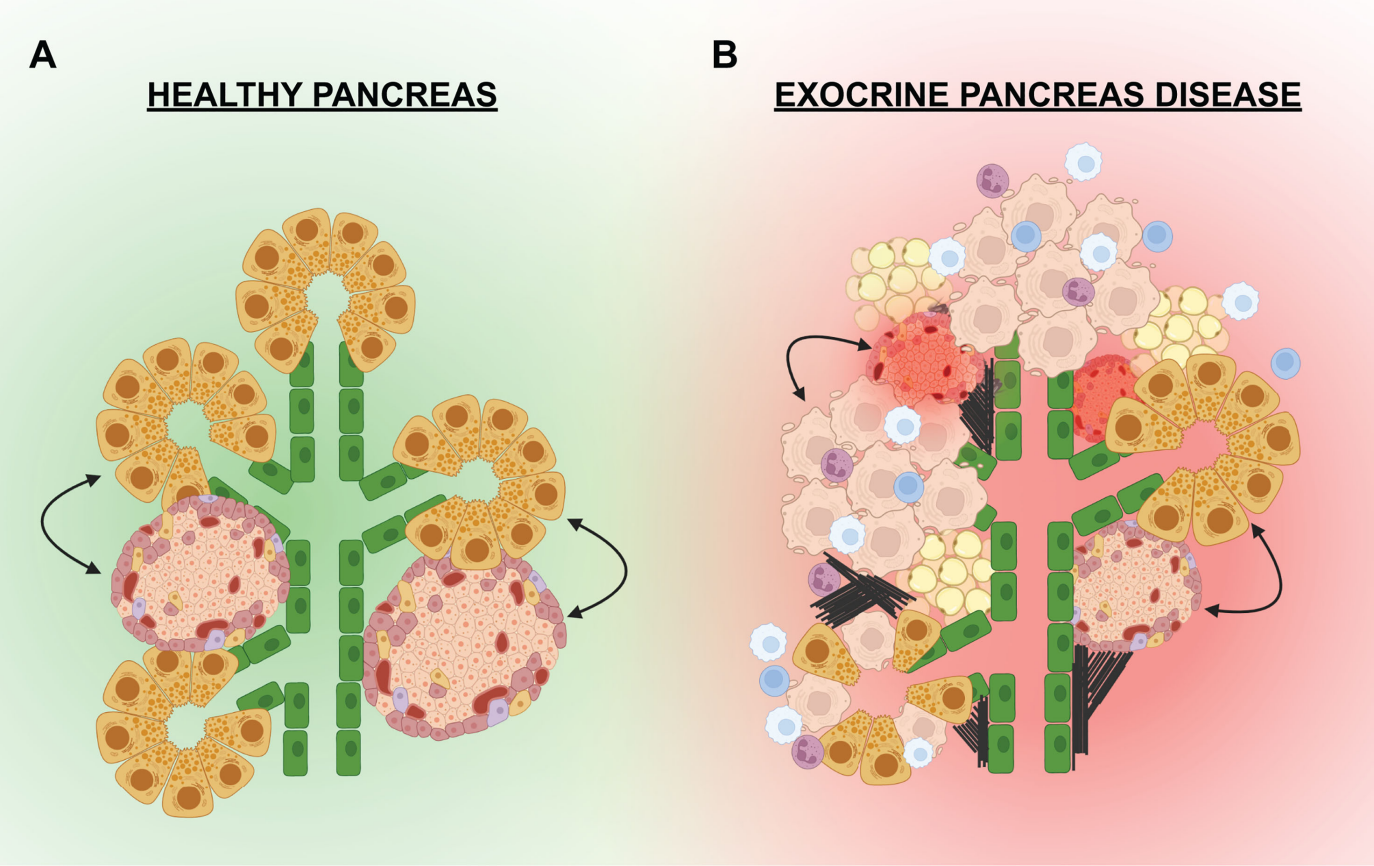
Exocrine-endocrine crosstalk in health and disease. **(A)** In a healthy pancreas, the islets are in physical proximity to the pancreatic epithelium (green) and the exocrine acinar cells (yellow). Paracrine factors or external cellular connections from the exocrine may influence the growth and function of the endocrine (and visa versa). **(B)** In the setting of exocrine disease, the pancreas can be infiltrated by immune cells (white, blue, purple) that promote inflammation and apoptosis of exocrine cells (beige). Fibrosis (black lines) and fatty deposits (tan) have also been noted in the pancreas of some exocrine diseases. Signals from the dying acinar cells, infiltrating immune cells, adipocytes, or external connections can negatively effect the islets leading to dysfunction and/or death.

“*Exocrine-endocrine crosstalk*” can be defined as the cellular communications i.e. paracrine signals and/or external connections from the exocrine pancreas that influence growth and function of the endocrine pancreas (and vice versa) (**Figure 3**). Whereas the endocrine and exocrine compartments are considered to be functionally distinct, there are direct and indirect connections between the compartments that promote cellular growth, differentiation, viability, and function. Investigating these connections may unearth exogenous factors that could stimulate beta cell growth, differentiation, or insulin secretion. For example, the inhibition of pancreatic elastase, which is produced by the exocrine acinar cells, has been shown to cause increased beta cell proliferation (62). Thus, evidence for direct communication between the two functional compartments of the pancreas is slowly coming to light. This direct communication is likely assisted by the physical proximity and interconnections that exist between the exocrine and endocrine compartments, which are established during embryonic pancreas development. As described above, the conclusion of pancreas development results in the formation of a structured organ wherein the endocrine cells begin to coalesce into islets, which are distributed throughout the exocrine pancreas. This architecture is likely supportive in a healthy state; however, in the setting of pancreatic disease and inflammation, the close proximity of islets to acinar cells often results in collateral damage and islet dysfunction.

### The Exocrine Pancreas in the Setting of Diabetes

Given the physical location of the islets within a sea of acinar cells in the pancreas, it is not surprising that pathological connections have been identified between the endocrine and exocrine pancreas. Both type 1 diabetes (T1D) and type 2 diabetes (T2D) have been described as having a high prevalence of exocrine insufficiency (63), with “exocrine insufficiency” being used as an umbrella term to describe an array of changes including general loss of pancreas size, acinar cell atrophy, and/or a reduction in exocrine enzymes (64–70). In addition to these changes, fibrosis, arteriosclerosis, and fatty infiltration have been observed in the pancreata of individuals with T1D (64). Furthermore, studies revealed that the clinical parameters of exocrine pancreas function including secretion of lipase, amylase, bicarbonate, trypsinogen, and fecal elastase, were decreased in individuals with T1D, which indicates the onset of exocrine insufficiency (64, 67, 68). Interestingly, the trend of reduced pancreas volume in T1D patients extends to first degree relatives, who also exhibit less total pancreas volume compared with healthy controls (66). Interestingly, individuals that express at least one T1D-associated autoantibody showed a similar trend of reduced pancreas volume compared with autoantibody negative controls, likely contributing to the increased risk of T1D (65). In studies examining pancreas size prior to the onset of T1D, it was revealed that the pancreas exhibits a decline in volume that begins with the appearance of autoantibodies and continues at least one year into clinical diagnosis (71). Of note, this study also determined that the reduced pancreatic mass was accompanied by a loss of structural integrity (71), which poses a possible explanation for decreased volume given that structural integrity is necessary for cell viability and proper function (72). What drives the loss of exocrine mass is not fully understood; however, there is data to support a mechanism that includes decreased exocrine cell number. Specifically, individuals with T1D displayed a striking 57% fewer total acinar cells compared with healthy controls (70). Interestingly, in this study the exocrine cell reduction could not be attributed solely to an increase in apoptosis, suggesting that there may be other factors that contribute to changes in pancreas size. Although the mechanisms driving exocrine loss and dysfunction remain unresolved, exocrine pancreatic insufficiency has become a significant characteristic associated with diabetes.

### Type 3c Diabetes

Direct evidence that endocrine function is impacted by exocrine dysfunction can be found in diseases wherein diabetes is observed following exocrine pancreas damage. Most significantly, Type 3c diabetes (T3cD) is a disease that begins in the exocrine pancreas due to pathologies such as chronic pancreatitis and pancreatic cancer; the disrupted exocrine pancreas then induces endocrine/beta cell dysfunction (73). This disease pathology underscores the impact of exocrine enzymes and other exocrine-associated proteins on islet and beta cell function. Studies suggest the loss of exocrine pancreas, as in chronic pancreatitis, contributes to T3cD indirectly by impacting insulin secretion *via* the incretin effect (73). Without nutrient absorption (mediated by pancreatic enzymes), the postprandial incretin response and insulin secretion are reduced, possibly impacting long-term beta cell health. Moreover, a study by Hostelley *et al.* (74) observed that exocrine pancreas-derived proteases regulate beta cell proliferation, specifically showcasing that an increase in certain pancreatic protease mRNAs correlated with an increase in beta cell mass. This would suggest that the changes in pancreatic enzyme levels observed in T3cD may be a confounding factor in beta cell health and progression to diabetes.

T3cD onset has also observed following pancreas cancer. Pancreatic ductal adenocarcinoma (PDAC) is a form of pancreatic cancer that originates from the epithelial cells of the pancreatic duct and results in the blockage of bicarbonate and enzyme secretion into the digestive tract (75, 76). PDAC has been reported to indirectly inhibit the secretion of insulin from pancreatic beta cells, which subsequently results in diabetes onset (77). The inhibition of beta cell secretion has been linked to tumor-derived exosomes that secrete micro-RNAs, which upregulate the PI3K/AKT/FOXO1 pathway, eventually leading to insulin resistance and beta cell failure (78). Interestingly, diabetes has been identified as a risk factor for pancreatic cancer, as later life diagnosis (after age 40) may predispose to PDAC (79, 80). Altogether, these studies point to a pathological connection between the endocrine and exocrine that influences pancreatic function. Exploring this connection may provide a greater understanding of the exocrine-derived factors and pathways that support proper endocrine health.

### Inherited Exocrine Disease

There exists clinical evidence from exocrine pathologies that exocrine-endocrine crosstalk can be a driver of disease. These include hereditary pancreatitis induced by mutations in *PRSS1*, metabolic syndrome caused by mutations in *CELA2A*, Cystic Fibrosis (CF), and Wolcott-Rallison Syndrome (81–84) (**Table 1**). In additional to genetic predisposition, these diseases may provide further evidence that diabetes onset following exocrine pancreatic disease stems from the physical proximity of the islets within a sea of dysfunctional acinar cells. Liu *et al.* (94) demonstrated that the prevalence of T3cD in patients with pancreatitis was increased in those patients with mutations in *PRSS1*, which cause improper production and secretion of the protease trypsinogen. This connection between diabetes onset and dysregulated protease production provides additional evidence that acinar cell secretions may play a role in the maintenance of beta cell health. Similarly, pancreatic elastase (encoded by *CELA2A*) is known to enhance insulin signaling by both promoting insulin secretion and mediating degradation (82). In the presence of *CELA2A* gene mutations and therefore nonfunctional CELA2A protein, dysregulated insulin secretion, hyperglycemia, and the eventual onset of T2D results (82, 95). Without this essential exocrine-derived protein, proper insulin signaling is unachievable. In essence, these studies identify factors that connect the exocrine pancreas function directly to islet health.

**Table 1:** Exocrine pathologies that progress to endocrine dysfunction.

Disease	Prevalence	Gene(s)	Pathologies & Symptoms	Diabetes incidence	References
Chronic Pancreatitis	50/100,000 people	N/A	Pancreatic inflammation and fibrosis Nutrient maldigestion and malabsorption Glucose intolerance Insulin deficiency	Up to 80%	(85, 86)
CFRD	In CF patients: 2% children 19% adolescents 40-50% adults	*CFTR*	Onset at 18-24 years old Microvascular complications Severe insulin deficiency Underweight	In CF patients: 35%	(87, 88)
Hereditary Pancreatitis	3/1,000,000 people	*PRSS1* *CELA2A*	Pancreatic inflammation and fibrosis Nutrient maldigestion and malabsorption Glucose intolerance Insulin deficiency Abdominal pain	*PRSS1*: up to 48% *CELA2A*: not yet determined	(82, 89, 90)
Wolcott-Rallison Syndrome	Extremely rare	*EIF2AK3*	Hepatic dysfunction Bone dysplasia Glucose intolerance Insulin deficiency	100%	(91)
MODY8	Rare	*CEL*	Nutrient maldigestion and malabsorption Glucose intolerance Insulin deficiency	100%	(92, 93)

#### Cystic Fibrosis-Related Diabetes

Cystic fibrosis related diabetes (CFRD) is a form of diabetes caused by the monogenic disease CF (96). CF is a disease that arises from mutations in the cystic fibrosis transmembrane conductance regulator (*CFTR*) gene, resulting in disruptions of bicarbonate and salt flux and an accumulation of mucus in exocrine glands (96). For individuals with CF, the incidence of CFRD is estimated to be as high as 35%, with anecdotal clinical data suggesting that the prevalence is steadily increasing (81). CFRD is commonly recognized as a pancreas injury-driven form of diabetes, as studies in both humans and animal models demonstrate that it occurs as a result of pancreatic inflammation and autolysis (97, 98). However, there is also evidence that CFRD occurs as a result of altered CFTR directly impacting general beta cell function (99–101), cAMP regulated exocytosis of insulin (100), and glucagon secretion (102). Given that not all individuals with CF develop CFRD raised the question as to the role of *CFTR* mutations in diabetes development. Interestingly, Hart *et al.* (103) demonstrated that the loss of *CTFR* in the pancreatic beta cells of mice *did not* alter oral glucose tolerance or islet insulin secretion. Therefore, this study refutes the claims that CTFR in islet cells is critical to beta and alpha cell function and supports the data that beta cell dysfunction occurs as an indirect result of pancreatic inflammation. When considered alongside the clinical observations from patients with CF, CFRD likely occurs as a consequence of the islet/beta cell location within a dysfunctional exocrine pancreas. Therefore, this disease underscores the influence of the surrounding environment on beta cell integrity and function.

#### Wolcott-Rallison Syndrome

Like CF, Wolcott-Rallison Syndrome is a rare genetic disorder in which one of the prevalent comorbidities is diabetes. Diabetes resulting from Wolcott-Rallison Syndrome manifests in early infancy and individuals with the disease often do not survive to adulthood (83). Interestingly, the origin of this disease has been linked to mutations in *EIF2AK3*, the gene that encodes PKR-like ER kinase (PERK), which functions in the Unfolded Protein Response (UPR) to ameliorate endoplasmic reticulum (ER) stress (104, 105). *EIF2AK3* expression levels are significantly higher in the pancreas (106, 107). Consistent with the importance of *EIF2AK3* in the pancreas, Harding *et al.* demonstrated that mice lacking PERK developed exocrine pancreatic dysfunction and diabetes due to increased ER stress in pancreatic secretory cells (108). Interestingly, this effect was most prominent in the beta cells and acinar cells. Studying monogenic diseases such as Wolcott-Rallison Syndrome, where disease consequences impact both the exocrine and endocrine, highlights the crosstalk between these cell types as well as the potential mechanistic connections between acinar cells and beta cells.

#### Maturity Onset Diabetes of the Young type 8

Maturity Onset Diabetes of the Young type 8 (MODY8) is a form of diabetes that results from mutations in Carboxyl Ester Lipase (*CEL*) (92). Secreted by acinar cells, CEL functions to breakdown fats and allow their proper absorption into the intestines [reviewed in (109)]. Mutations in the *CEL* gene result in exocrine pancreatic insufficiency and eventual progression to diabetes. The mechanism underlying the progression to diabetes is not entirely clear; however, exocrine-endocrine crosstalk has been implicated. Kahraman et al. (110) demonstrated that *CEL* mutations produce dysfunctional CEL that is endocytosed by beta cells. Upon internalization, mutant CEL forms aggregates that disrupt beta cell secretion, ultimately hindering the beta cell’s capacity to maintain whole-body glucose metabolism. In essence, this supports the idea that exocrine pancreatic enzymes directly influence beta cell function and adds to the list of clinical diseases that provide evidence for proper endocrine/beta cell function being dependent upon proper acinar cell function.

### Secreted Factors Can Drive Exocrine-Endocrine Crosstalk

In addition to physical proximity and the indirect effects from organ dysfunction, there is evidence that crosstalk between cells of the exocrine and endocrine pancreas can be facilitated by endogenous factors. In fact, the direct effects of exocrine secretions on beta cell health, function, and longevity have become a focus of significant investigation. Serine protease inhibitors (Serpins) and pancreatic enzymes such as carboxyl ester lipase (CEL), have been identified to influence beta growth and function, demonstrating another avenue through which the exocrine pancreas can exert effects on the endocrine. In particular, SerpinB1 is a pancreatic elastase inhibitor shown to promote beta cell proliferation by modulating growth and survival signaling (62). Derived from the liver, this inhibitor promotes the proliferation of beta cells in zebrafish, mice, and humans, indicating a general rather than organism-specific role (62). SerpinB1 inhibits pancreatic elastase, which suggests that in the normal setting certain acinar cell-derived digestive enzymes negatively impact beta cell growth, indicating direct exocrine-endocrine communication. Similar to SerpinB1, SerpinB13 influences beta cell growth by inhibiting a pancreatic protease. Specifically, SerpinB13 inhibits cathepsin L, known for its prominent role in pancreatic cancer metastasis; it is stored in and secreted from pancreatic lysosomes and ultimately functions by aiding in the modification of the extracellular matrix (111). Kryvalap *et al.* (112) demonstrated that the presence of SerpinB13 antibodies promoted beta cell development and imparted resistance to T1D. This occurred as a result of increased Notch1 cleavage leading to an upregulation in Notch signaling and growth (112). Excitingly, a decrease in SerpinB13 has also been found to be clinically relevant. Children with T1D who are given SerpinB13 antibodies showed a slower decline in residual beta cell function, which demonstrated the therapeutic potential for targeted protease inhibition (113). Taken together, these studies support the continued investigation of endogenous exocrine secretion in the promotion of beta cell growth and function. Moreover, the communication between acinar cells and beta cells *via* paracrine signals, physical connections, or environmental consequences of dysfunction demonstrates the importance of “exocrine-endocrine crosstalk” on beta cell health.

## Discussion

Cell-cell communication can influence organ development, function, and disease. Physical and molecular interactions between the cells in the pancreatic islet are known to facilitate and support beta cell function (**Figure 2**). However, the communication between exocrine and endocrine cells and the influence of this signaling on growth and function is not well understood. There is a growing body of literature from studies of both development and disease that suggest islets/beta cells are influenced by the surrounding pancreatic exocrine acinar cells. The study of “exocrine-endocrine crosstalk” (**Figure 3**) has therefore become a burgeoning area of investigation in the diabetes research field.

The need to decipher the mechanisms that drive intra-pancreatic cell communication and the influence of this crosstalk on cell and organ function has been driven by clinical observations. In particular, the occurrence of endocrine dysfunction, specifically diabetes, after exocrine pancreas damage or loss is significantly observed in many diseases (**Table 1**). Conversely, donor pancreas tissue from individuals with type 1 diabetes has been analyzed and shows a reduction in exocrine pancreas weight, acinar cell number, and exocrine-derived enzyme levels (63–70). Despite these convincing clinical observations supporting the idea of crosstalk, it remains unknown if the observed impact on organ function and size results from direct, targeted, cell-cell communication or simply occurs as collateral damage from disease. As noted above, the study of the origin of beta cell dysfunction in CFRD epitomizes this debate. There is research suggesting that *CFTR* loss-of-function drives impaired insulin secretion (99, 100); however, there is also evidence that the resultant diabetes occurs solely as a consequence of pancreatic inflammation (103, 114). Whether there are signals directly from the dysfunctional/dying exocrine cells that influence beta cell function remains a critical gap in knowledge.

Understanding the environment necessary for beta cell survival and proper insulin secretion is necessary for the generation of effective and long-lasting diabetes therapies. Current research demonstrates that islet transplantation is a successful therapy (115, 116). However, given the mounting evidence that the exocrine pancreas provides critical support and possibly direct maintenance of islet health, the question could be posed as to whether there would be even greater success if donor islets were transplanted into a more “pancreas-like” environment instead of the liver. The liver is clearly devoid of acinar cell and therefore the islets do not receive the expected endogenous exocrine signals. Perhaps donor islet health could be maintained for a greater period of time if transplanted into a more native environment or if whole pancreas transplant were performed. Clearly, further investigation is needed to fully understand the interactions between beta cells and exocrine tissue, and how these interactions might positively benefit islet transplant protocols.

To that end, the study of “exocrine-endocrine crosstalk” has produced novel findings related to the influence on pancreatic cell growth. In particular, studies of exocrine-derived secreted factors revealed that certain digestive enzymes can influence beta cell growth. For example, the inhibition of pancreatic elastase was found to directly stimulate beta cell growth (112). This finding alone has sparked excitement for the development of therapies; however, it is not without potential caveats. The mechanisms that drive beta cell expansion after treatment with digestive enzymes or enzyme inhibitors remains a gap in knowledge. It will be critical to understand the mechanism of action driving pancreatic enzymes to stimulate beta cell growth in the disease setting. As developmental mechanisms can often be coopted by diseased cells to drive pathology, we could look to the study of normal pancreatic development (**Figure 1**) to determine if exocrine enzymes serve a critical function during organ formation. It may be that exocrine signals (possibly in the form of secreted enzymes) function to restrict islet cell growth during development and, thus, inhibiting these enzymes in either a healthy or diseased state would stimulate beta cell growth. Altogether, defining the factors that direct beta cells to thrive, the influence of the exocrine on their differentiation, and the intra-pancreatic signals that promote their proper function may reveal new avenues for the development of therapeutics that can reverse diabetes.

## Author Contributions

All authors listed have made a substantial, direct, and intellectual contribution to the work and approved it for publication.

## Funding

Funding provided by a Juvenile Diabetes Research Foundation (JDRF) Career Development Award (5-CDA-2016-194-A-N), an NIH R01 (1R01DK121987-01A1) and NIH R01 supplement (R01DK121987-S1) to TLM.

## Conflict of Interest

The authors declare that the research was conducted in the absence of any commercial or financial relationships that could be construed as a potential conflict of interest.

## Publisher’s Note

All claims expressed in this article are solely those of the authors and do not necessarily represent those of their affiliated organizations, or those of the publisher, the editors and the reviewers. Any product that may be evaluated in this article, or claim that may be made by its manufacturer, is not guaranteed or endorsed by the publisher.
